# Modulation of the gut–brain axis via the gut microbiota: a new era in treatment of amyotrophic lateral sclerosis

**DOI:** 10.3389/fneur.2023.1133546

**Published:** 2023-04-20

**Authors:** Du Hong, Chi Zhang, Wenshuo Wu, Xiaohui Lu, Liping Zhang

**Affiliations:** ^1^The First Affiliated Hospital of Zhejiang Chinese Medical University, Hangzhou, China; ^2^Sir Run Run Shaw Hospital, Zhejiang University, Hangzhou, China

**Keywords:** gut microbiota, gut-brain axis (GBA), intestinal barrier, autophagy, probiotics

## Abstract

There are trillions of different microorganisms in the human digestive system. These gut microbes are involved in the digestion of food and its conversion into the nutrients required by the body. In addition, the gut microbiota communicates with other parts of the body to maintain overall health. The connection between the gut microbiota and the brain is known as the gut–brain axis (GBA), and involves connections via the central nervous system (CNS), the enteric nervous system (ENS), and endocrine and immune pathways. The gut microbiota regulates the central nervous system bottom-up through the GBA, which has prompted researchers to pay considerable attention to the potential pathways by which the gut microbiota might play a role in the prevention and treatment of amyotrophic lateral sclerosis (ALS). Studies with animal models of ALS have shown that dysregulation of the gut ecology leads to dysregulation of brain–gut signaling. This, in turn, induces changes in the intestinal barrier, endotoxemia, and systemic inflammation, which contribute to the development of ALS. Through the use of antibiotics, probiotic supplementation, phage therapy, and other methods of inducing changes in the intestinal microbiota that can inhibit inflammation and delay neuronal degeneration, the clinical symptoms of ALS can be alleviated, and the progression of the disease can be delayed. Therefore, the gut microbiota may be a key target for effective management and treatment of ALS.

## 1. Introduction

Amyotrophic lateral sclerosis (ALS) is a neurological disorder of unknown etiology involving the motor neurons, usually accompanied by degeneration of the associated neurons. It is the most common neurodegenerative disease of middle age, with a lifetime risk of occurrence of one in 300 (1) and a peak age of onset of ~60 years (2). ALS leads to progressive paralysis with reduced speech, swallowing, and motor function, eventually resulting in respiratory insufficiency and death. The median survival time of ALS patients is ~2–4 years, but this varies widely, with some individuals surviving for more than 50 years. It has been estimated that ALS causes ~30,000 deaths worldwide each year (3). Factors influencing survival heterogeneity may be environmental or genetic, and some may be random. The etiology of ALS is still unclear, but the more widely accepted factors include genetics, oxidative stress, excitotoxicity, neurotrophic factor disorders, autoimmune mechanisms, viral infections, and environmental factors (4). Approximately 10% of ALS cases are family aggregates (fALS), which can be transmitted within families, and the rest are considered sporadic episodes (sALS) (5) with no clear family history. The incidence of ALS varies slightly by gender: the risk of developing the disease is slightly higher among men (3.0 cases per 100,000 person-years) compared to women (2.4 cases per 100,000 person-years). However, for fALS, the risk is similar for both men and women. The majority of patients (~70%) present with limb-initiating ALS, ~25% present with bulbar onset, and the remainder (5%) present with respiratory involvement or initial trunk onset (6). There is no specific treatment available for ALS, although the more popular novel treatments include gene therapy (7) and stem cell therapy (8). However, two recognized therapeutic agents have been classified as disease-modifying drugs, with riluzole being the most widely used (6). Despite its ability to slow the progression of ALS, its effectiveness is limited. Another drug, edaravone, has also been approved by the FDA for oral use (9) and possesses potent antioxidant properties that mitigate the effects of oxidative stress by eliminating oxidized lipids and hydroxyl radicals, which may be a key factor in the pathogenesis and development of ALS. However, clinical studies have also suggested that edaravone may be ineffective in treating ALS (10). Other drugs include phenylbutyrate-taurursodiol (11) (sodium phenylbutyrate and urocholine). Despite these therapeutic options, there is currently a lack of effective treatments for ALS; therefore, it is crucial to seek a novel approach to treatment.

The gastrointestinal tract contains hundreds of millions of microorganisms, collectively referred to as the gut microbiota, which are formed according to host genetics and environmental exposure. The gut microbiota includes bacteria, fungi, pathogens, commensals, viruses, protozoa, and parasites (12), with both disease-causing and disease-preventing organisms among them. In a healthy human gut, these microorganisms play a crucial role in maintaining overall health and in the development of diseases. Moreover, research has identified links between diseases whose etiology is not yet clear and the gut bacterial map (13, 14). The gut microbiota plays a role in human metabolism (including food and drug metabolism); gut homeostasis; maintenance of the integrity of the gut barrier; immune development, immunomodulation, and prevention of pathogens; and brain processes and behavior (15, 16). When the gut microbiota undergoes pathological changes, its metabolites also change, leading to corresponding changes in normal human physiological functions through immune and circulatory mechanisms, thereby affecting the development of disease (17). Conversely, disease in turn also affects the gut, leading to changes in the gut microbiota. The intestinal microbiota has been found to be a unique ecosystem and has been referred to as the “second genome” of humans (13). With the widespread use of next-generation sequencing (NGS) (18), especially 16SrRNA and shotgun sequencing, the relationship between the gut microbiota and various systemic diseases has been gradually revealed. Lack of a “healthy” microbiota is associated with many forms of disease (19–24), such as metabolic diseases (obesity, type 2 diabetes), gastrointestinal cancer, inflammatory bowel disease, and renal disease, as well as neurodegenerative diseases (NDs) associated with aging, in which the activity and composition of the gut microbiota change during the life cycle. Fang et al. (25) found that altering the gut microbiota in animal models alters immune function, metabolic activity, and ultimately lifespan by mechanisms associated with the aging process, and that alterations to the gut microbiota may positively or negatively affect the neuropathology and clinical manifestations of disease. A growing number of studies have reported changes in the microbiota among patients with Parkinson's disease (PD), Alzheimer's disease (AD), ALS, and Huntington's disease (HD) (26–28), and although the exact mechanisms underlying these phenomena remain unclear, there is a growing body of evidence to support the concept of a “gut microbiota–brain axis” (29). Overall, these findings have generated interest among researchers in further investigation of the microbiota-gut-neural system.

ALS is a fatal neurodegenerative disease of the nervous system. Numerous studies have revealed an inextricable relationship between translocation or altered abundance of gut microbes and risk factors for the onset and progression of ALS, as well as its treatment and prognosis (30–32). Studies have found that, in ALS patients, gastrointestinal symptoms precede neurological symptoms in all cases (33); these include gastroesophageal reflux, chronic constipation, intermittent diarrhea, bloating, and abdominal pain. The gut–brain axis (GBA) consists of the CNS, the autonomic nervous system (ANS), the endocrine system (ENS), the hypothalamic-pituitary-adrenal (HPA) axis, and the immune system (34). The GBA is a bidirectional communication pathway between the gut and the CNS, involving multiple feedback loops and the interaction of different channels (34). The gut microbiota is closely linked to the CNS through the GBA (34). Existing evidence suggests that bottom-up regulation of the CNS by the gut microbiota occurs primarily through neuroimmune and neuroendocrine mechanisms, mediated by microbial-derived molecules such as short-chain fatty acids (SCFAs), secondary bile acids (2BAs), and tryptophan metabolites, which propagate signals primarily through interactions with enteroendocrine cells (EECs), enterochromatic cells (ECCs), and the mucosal immune system, with some molecules crossing the intestinal barrier, entering the systemic circulation, and possibly crossing the blood–brain barrier (BBB) (35). The ANS can transmit afferent and efferent nerve signals between the gut and the brain (35). The HPA axis is an important component of the neuroendocrine system (36). On the one hand, in pathological states in which gut bacteria are displaced and their abundance is altered, the intestinal microbiota produces pro-inflammatory cytokines such as lipopolysaccharides (e.g., endotoxin LPS) during the processes of composition and catabolism, and these affect the synthesis of γ-aminobutyric acid (GABA), serotonin, and glutamate, ultimately leading to the development of neurological inflammation (37). On the other hand, considering the role of the GBA, the emergence of an unhealthy gut microbiota can alter intestinal permeability and cause a series of problems. First, changes in intestinal permeability can easily cause damage to the intestinal barrier, resulting in increased permeability of the intestinal epithelium and, to a certain extent, decreased expression of intestinal junction proteins. Second, when the intestinal barrier is destroyed, inflammatory factors (such as microglia, inflammasomes, complement proteins, and cytokines) (38, 39), unhealthy intestinal microflora, and toxic substances become able to enter the circulatory system, activating the intestinal neuroimmune system, thereby destroying neuronal cells and affecting the onset and development of ALS (35, 36).

## 2. Bidirectional communication between the gut microbiome and the brain

**Figure 1:**
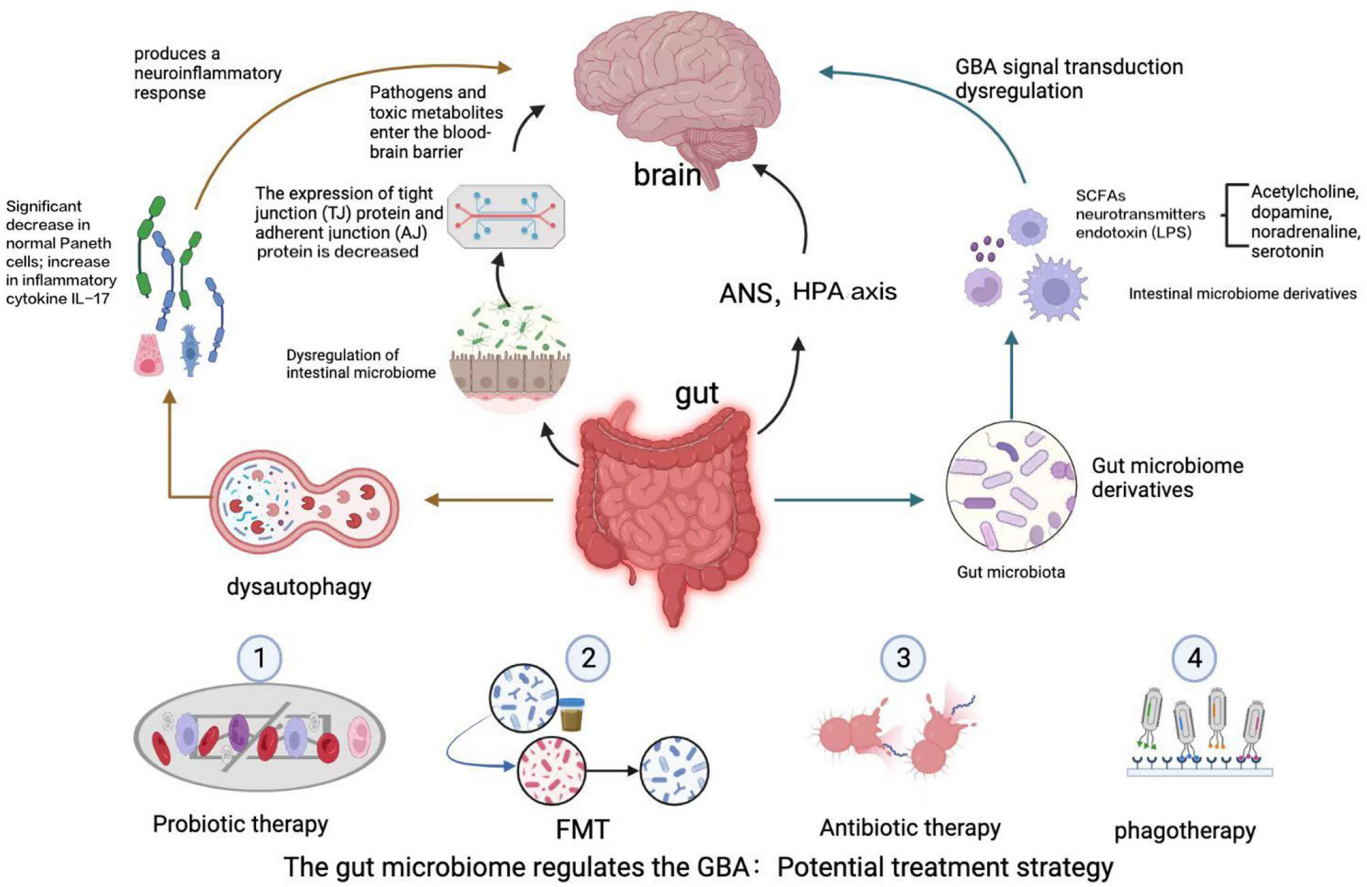
The GBA is a bidirectional communication pathway between the gut and the CNS involving multiple feedback loops. Changes in the gut microbiota lead to alterations in the intestinal epithelial barrier, and the gut microbiota produces a variety of neuroactive metabolites, including SCFAs, neurotransmitters, and endotoxins. These factors affect autophagy, activate an immune response, and lead to dysregulation of the GBA via the ascending pathway from the gut to the brain, triggering neuroinflammation and neurodegenerative diseases of the central nervous system, such as ALS, AD, and PD. A potential avenue for treatment of ALS may involve manipulation of the gut microbiota and its derivatives, including via intestinal mucosal resurfacing, dietary intervention, probiotic supplementation, antibiotics, and post-antibiotic therapy, among other potential methods.

Bidirectional communication in the GBA occurs through multiple complex pathways. Although the precise pathways by which gut microbes communicate with the CNS through the GBA remain to be determined, both direct pathways (including the ENS and the vagus nerve) and indirect pathways (involving neurotransmitters, SCFAs, and cytokines) are known to be involved. The sympathetic and parasympathetic nervous systems and the vagus nerve, in turn, affect the CNS through the production of bacterial metabolites (40). The specific process is shown in Figure 1.

### 2.1. The gut microbiota and the HPA axis

The HPA axis, or neuroendocrine system, is an important component of the GBA (36); its role involves the transmission of information mainly through the vagus nerve. The neuroendocrine system, which is involved in controlling stress responses, consists primarily of the vagus nerve and sensory neurons that transmit information from the gut to the brain stem, which, in turn, includes the hypothalamus and the limbic system. Under stress stimulation, the limbic system in turn affects the autonomic activities of the intestine (41). In the GBA, responses to stressful stimuli (including digestion, immune system function, mood, and energy balance) are mediated through afferent and efferent nerves that in turn activate the HPA axis (41). The hypothalamus releases an adrenocorticotropin-releasing hormone (CRH), which reaches the pituitary gland through the bloodstream and stimulates the synthesis of adrenocorticotropic hormone (ACTH). ACTH stimulates the synthesis of glucocorticoids (stress hormones), such as cortisol or corticosterone, in the adrenal glands, resulting in a systemic response that leads to leakage from the tight junctions of the intestine, thus increasing the permeability of the intestinal barrier (41–43). Moreover, excessive release of cytokines (IL-1β, IL-6, and TNF-α) and small bioactive molecules, as well as gut microbial derivatives such as lipopolysaccharide (LPS) and peptidoglycan (44), means that these can cross the BBB into the brain and activate the HPA axis when there are changes in the diversity and abundance of the gut microbiota (42). These findings suggest that communication between the gut microbiota, the neuroendocrine system, and the brain occurs through multiple direct and indirect pathways; further exploration of the mechanisms involved is warranted.

### 2.2. The gut microbiota and the ENS

The GBA is the main communication pathway between the ENS and the CNS (45). The ENS is responsible for coordinating intestinal functions such as intestinal motility, fluid secretion, and metabolite responses to gut microbiota production. Evidence currently suggests that specific bacterial strains may play a key role in the development and function of the ENS (43). The gut microbiota interacts with the ENS via the vagus nerve (46) and plays an important role in regulating the gastrointestinal tract, influencing the development and function of the ENS and enteroglial cells (EGCs). The gut microbiota also communicates with the CNS through direct pathways [via regulation of gut neuron survival and promotion of neurogenesis (47)] and through indirect pathways involving neurotransmitters (e.g., serotonin), bacterial metabolites (e.g., SCFAs), and cytokines (48, 49). In response to signals from the gut, the CNS sends regulatory messages to the intestinal microenvironment via ENS units of neuron–glial–epithelial cells, regulating the contraction of intestinal smooth muscle and the activity of glands and blood vessels (50). Gut microbes are key regulators of GBA function and modulation of neuronal activity via stimulation of the intestinal ENS (50).

### 2.3. Neuroactive metabolites derived from the gut microbiota

#### 2.3.1. SCFAs

The brain and the gastrointestinal tract are key sensory organs responsible for detecting, transmitting, integrating, and responding to signals from the *in vivo* and *in vitro* environment (51, 52). SCFAs are important metabolites of the gut microbiota, produced by the anaerobic fermentation of indigestible polysaccharides (e.g., dietary fiber and resistant starch) in the colon, and consist mainly of acetate (40%−60%), propionate (20%−25%), and butyrate (15%−20%) (53). The gut microbiota of the *Bacillus* produces acetate and propionate, and individuals of this thick-walled phylum produce large amounts of butyrate. SCFAs are mostly in ionized form and require transporter proteins for absorption. In the small intestine, transport of SCFAs is effected by monocarboxylate transporter protein (MCT)1 (SLC16A1), sodium-coupled MCT (SMCT)2 (SLC5A12), and SLC16A7; and in the colon, this function is fulfilled by MCT1 (SLC16A1), SMCT2 (SLC5A12), SMCT1 (SL5CA8), and SLC26A3. Therefore, the concentration of SCFAs depends on absorption and local physiological concentration (7). It has been shown (54) that the gut microbiota can regulate genes through metabolism, differentiation, and proliferation, and that butyrate regulates the expression of 5%−20% of human genes. SCFAs can help maintain the integrity of the intestinal epithelial barrier and the innate tolerance of intestinal immunity by regulating epithelial cell growth and differentiation and by affecting protein expression in tight junctions and mucosal permeability (55). They can also cross the BBB by upregulating the expression of tight junction proteins in the BBB with the assistance of endothelial cell monocarboxylate transport proteins (56). Through SCFAs, the gut microbiota actively communicates with host cells to regulate neurotrophic factors, neurotransmitters, and levels of neuroinflammation by affecting glial cell morphology and function, mitochondrial function, immune activation, lipid metabolism, and gene expression, thereby influencing CNS functions. In addition, various effects on host metabolism and the immune system have been observed (54, 57). For example, SCFAs (mainly butyrate) modulate the systemic inflammatory response by inducing T regulatory cell (Treg) differentiation and interleukin secretion. A significant elevation in the number of T cells was observed when germ-free (GF) mice were treated with SCFAs (58). Xiao et al. (59) found that modulation of the gut microbiota and supplementation with SCFAs promoted gastrointestinal transit, and modulated intestinal barrier function in a rat model of bilateral carotid artery obstruction, as well as improving symptoms of cognitive decline and depressive-like behavior, revealing the neuroprotective potential of gastrointestinal microbes. As this evidence shows, gut microbial metabolites (SCFAs) can exert a wide range of beneficial effects on the host and have been implicated in the prevention and treatment of many diseases. SCFAs affect GBA signaling and may directly or indirectly influence the pathophysiology of NDs (29).

#### 2.3.2. Neurotransmitters

Johnson and Foster (60) found that the different microbes of the gut microbiota secrete different neurotransmitters: e.g., *Bacillus* species secrete mainly acetylcholine, dopamine, and norepinephrine, while *Escherichia* secrete serotonin. The intestinal microbiota and its metabolites (e.g., SCFAs) can also produce serotonin, norepinephrine, and GABA through stimulation of intestinal chromogranin cells (52), and these neurotransmitters can reach the brain and directly affect central nervous system function through the intestinal epithelium or the BBB (24). Furthermore, we found that the overall proportions of various neurotransmitters are higher in the gut than in the brain (39), and Bellon et al. (61) found that enterochromaffin cells synthesize more than 90% of serotonin synthesized *in vivo*, this being a key neurotransmitter involved in regulation of mood, feeding, sleep, and pain processing (62). El Oussini et al. (63) found that, in mice with ALS-linked SOD1 mutations, the conditional allele of SOD1 (G37R) caused degeneration of serotonergic neurons, leading to the inactivation of 5-HT2B/C receptors, with clinical manifestations such as increased muscle tone and spasticity disorders. Additionally, in a mouse model, elective deletion of mutant SOD1 expression was found to maintain the serotonin innervation of spinal motor neurons to a large extent, alleviating the manifestations of increased muscle tone.

#### 2.3.3. Endotoxins

The gut microbiota releases byproducts from the cell wall, such as endotoxins like LPS, which can act locally and may be present in the gut, gingiva, and skin, as well as other tissues, causing dysregulation of GBA signaling (64), or can cross the intestinal barrier to enter the circulatory system; this is called metabolic endotoxemia (65). LPS is the organism's innate immune-sensing “alarm molecule” that elicits a host immune response (66). Alhasson (67) observed simultaneous intestinal inflammation and neuroinflammation in a mouse model of Gulf War disease, suggesting that alterations in the microbiota may lead to leaky gut and endotoxemia, exacerbating neuroinflammation and activation of Toll-like receptors (specifically, TLR4) in the small intestine and brain. TLR4 (68) is an innate mammalian receptor central to the immune system and plays a key role in LPS-mediated inflammatory responses by recognizing LPS, activating the TLR4 signaling pathway, releasing pro-inflammatory factors, and inducing inflammatory responses. Toll-like receptors TLR2 and TLR4 have recently been found to be expressed at higher levels in both the blood and the brain in patients with Parkinson's disease (69). Elevated blood LPS levels have been found in ALS patients (51), which may be attributable to gut inflammation and microbiota changes (64). Although the question of whether neuroinflammation is a cause or a consequence of ALS remains to be investigated, what can be stated is that widespread and prolonged neuroinflammation exacerbates the degeneration of motor neurons (70). TDP-43 is an ALS-specific mutated gene, and the addition of LPS to microglia or astrocytes in culture can cause improper localization and aggregation of TDP-43. The addition of peripheral LPS in TDP-43 (A315T) transgenic mice can lead to TDP-43 aggregation *in vivo* (71). This suggests that increased endotoxin levels, combined with TDP-43 aggregation, exacerbate neurodegeneration and are relevant to the pathogenesis of ALS.

## 3. Dysbiosis of the gut microbiota in ALS affects the GBA

### 3.1. Impaired intestinal barrier due to dysbiosis of the intestinal microbiota in ALS patients

The gut microbiota and its metabolites, such as SCFAs, contribute to protection of the integrity of the intestinal epithelial barrier. Studies have shown (24) that pathological changes in the composition of the intestinal microbiota can lead to disruption of the intestinal epithelial barrier and increased mucosal permeability. As well as inducing intestinal motor dysfunction, this disruption of the integrity of the barrier can also lead to dysregulation of the GBA via the ascending tracts of the gut–brain pathway, triggering neuroinflammatory and neurodegenerative conditions of the central nervous system, such as ALS, AD, multiple sclerosis (MS), and PD. A study by Wu et al. (48) found that an ALS-specific SOD1^G93A^ mouse model exhibited dysregulation of the intestinal ecology (characterized by a reduction in the abundance of *Vibrio fibrate butyric acidophilus, Escherichia coli*, and *thick-walled phylum*) early in the disease progression, before the onset of neuromuscular symptoms. These changes were associated with reduced expression of tight junction (TJ) and adherent junction (AJ) proteins, as well as impairment of the intestinal barrier, leading to increased intestinal permeability. A permeability analysis of mouse sera revealed a two-fold increase in the fluorescence intensity readings of immunofluorescence-labeled FITC-dextrin in SOD1^G93A^ mice compared to wild-type mice, further indicating altered intestinal integrity and impairment of the intestinal barrier in SOD1^G93A^ mice. In addition, the number of abnormal Paneth cells and the level of pro-inflammatory IL-17 cytokines were increased in the intestinal tissues and blood of ALS mice. Zhang et al. (72) found, using the 16sRDNA technique, that butyrate-producing bacteria (*Butyrivibrio Fibrisolvens*) were reduced in SOD1^G93A^ mice, but after butyrate treatment, the mice exhibited restoration of homeostasis of the intestinal flora, restoration of intestinal epithelial barrier integrity, and improvement in clinical signs. It can be hypothesized that damage to the intestinal barrier is correlated with the development of ALS.

### 3.2. The gut microbiota and genetics in patients with ALS

Interaction between the host genome and the gut microbiota (53) affects the normal signaling of the GBA, and gene variants can regulate the gene-editing system of the natural gut flora and influence the enterobacterial composition. Significant progress has been made in identification of the genetic abnormalities associated with ALS, with at least 20 genes having been confirmed to be associated with the condition. The most common genes identified in clinical practice include SOD1, C9ORF72, TARDBP, FUS, and OPTNTD-P43 (55–57, 73, 74). Genetic variation activates phages that originally coexisted naturally with intestinal microbial hosts. Phages can cause immune disorders by recognizing foreign genetic elements and destroying their DNA fragments (75). Chronic neuroinflammation caused by immune disorders triggers the accumulation of misfolded proteins in nervous system cells and peripheral nerves (76), further leading to neuronal death (77). Aggregation of the now well-established SOD1 mutant protein is a hallmark of ALS pathology (78), and SOD1^G93A^ mice (containing the human Cu/Zn superoxide dismutase gene mutation SOD1^G93A^) can serve as a model for the neuronal and muscle damage that occurs in human ALS. Intestinal biolysis and increased intestinal permeability have been observed in the SOD1^G93A^ ALS mouse model (79). Zhang et al. (80) found that SOD1^G93A^ mice exhibited changes in the intestinal microbiota at 1 month and structural defects in the intestinal nervous system starting from 2 months. The accumulation of SOD1^G93A^ in the spinal cord and neurons serves as a molecular marker of the progression of ALS in SOD1^G93A^ mice, beginning at 2 months of age; the aggregation of human SOD1^G93A^ protein is observed in the white and gray matter of the lumbar spine, accompanied by increased levels of glial fibrillary acidic protein (GFAP) and neuromuscular symptoms such as decreased muscle strength, which are indicative of neuromuscular degeneration. Additionally, disruption of GFAP in the ENS suggests that the gut microbiota affects gut neuromuscular structure as well as function, and that imbalance in the gut microbiota may act as a potential trigger for degenerative diseases (81). Further research is needed to fully understand this relationship. The association between gut flora and ALS was revealed in a 2022 genome-wide association study (GWAS) of ALS (82), which analyzed data from 20,806 patients and 59,804 control individuals. *Enterobacteriaceae* and unclassified *Acidaminococcaceae* were found to be associated with a higher risk of ALS. Additionally, γ-glutamyl amino acids may be negatively associated with the risk of developing ALS, while γ-glutamyl phenylalanine is a specific risk factor for the disease. Both of its metabolites, 1-arachidonoyl-GPI and 3-methyl-2-oxobutyrate, have been found to increase the risk of developing ALS, whereas increased levels of 4-acetylaminobutyric acid may reduce the risk of ALS. Nezami and Srinivasan (83) found that transgenic mice with human ALS mutations (Prp-TDP43 A315T58) exhibited defects in the intestinal neurons, which may be associated with genetic mutations that result in abnormalities of the microbiota and the mucosal barrier. Another mouse model study (30) indicated that ALS-prone Sod1 transgenic (Sod1-Tg) mice exhibit significant mitigation of the decrease in intestinal motility as a result of improvement of the intestinal flora: specifically, *Akkermansia muciniphila* (AM) was found to alleviate symptoms of ALS, whereas *Ruminococcus torques* and *Parabacteroides distasonis* were found to exacerbate them. Studies of genetically mutated C9ORF72 mice have shown that mutations in C9ORF72 cause infiltration of neutrophils and other immune cells and activation of microglia in the spinal cord (79). A proinflammatory feeding environment leads to a decrease in the alpha diversity of the gut microbiota. When broad-spectrum antibiotics are used to reduce the burden of intestinal microbes, this phenotype is improved, and the lifespan of such mice is prolonged (74).

Notably, many severe neurodegenerative diseases, such as ALS, AD, and PD, are difficult to treat with conventional drugs. Neurodegenerative alterations are caused by a complex combination of genetic, environmental, and transgenic interactions, which are more closely linked than previously believed (84). There is a need for further elucidation of the effects of gene editing on the CNS, bottom-up through the GBA, via regulation of the gut microbiota.

### 3.3. Gut microbiota dysbiosis and autophagy in ALS patients

The gut microbiota has been shown to play a fundamental role in deeply connecting the gut to the brain. Clinical and experimental evidence (48) suggests the presence of biological barriers to the gut microbiota and microbial metabolites in ALS. Of particular interest among the proteostasis pathways that have been characterized to date is the process known as autophagy (85). This self-consumptive pathway maintains cellular and organismal integrity in to the context of various developmental events or stressors by breaking down cellular components and organelles for reuse or redistribution. Chua et al. (85) have shown that autophagy and proteostasis are not only observed in experimental models of fALS, where specific genetic mutations can impair autophagic effectors or generate proteotoxic stress, but may also play a key role in the pathogenesis of sALS. Metabolites of the intestinal microbiota regulate intestinal inflammation through the autophagic pathway. In addition, autophagy in turn breaks down invading pathogens, regulates pathogens, triggers the release of pro-inflammatory cytokines, and participates in antigen presentation and lymphocyte development (25). Dysregulated autophagy mediated by dysbiosis affects the integrity of the intestinal barrier and increases intestinal permeability, thereby translocating intestinal microbes, their metabolites, and microbial-associated molecular patterns to mesenteric lymphoid tissue. The resulting neuroinflammatory response causes the development and progression of neurological disorders. Dysregulated autophagy leads to impaired clearance of polyubiquitinated protein aggregates in neurons and impairs endoplasmic reticulum (ER) homeostasis during stress, in aging, and in neurodegenerative diseases such as ALS. These findings suggest that the failure of the autophagic system to clear misfolded proteins is a major trigger of neuroinflammation and is closely associated with the development of ALS (86), in which inflammation propagates from somatic circulation to the brain via the cerebro-intestinal axis; they also suggest that protein misfolding and its aggregation, axonal damage, and neuronal demyelination are among the main mechanisms leading to neurodegenerative lesions. Wu et al. (48) found that the intestines of SOD1^G93A^ mice (with impaired tight junction structures) exhibited increased permeability, significantly reduced expression levels of tight junction protein ZO-1 and adhesion junction protein E-cadherin, significantly reduced normal Paneth cells, and increased levels of inflammatory cytokine IL-17. Paneth cells are epithelial cells in the small intestine that regulate intestinal autophagic activity and host–bacteria interactions (38). Paneth cells contain antimicrobial peptides that play an important role (87) in the host's innate immune response and in shaping the gut microbiome. Dysregulation of autophagy appears to lead to the elimination of misfolded proteins, such as superoxide dismutase 1, in G93A mice, and this dismutant form accumulates in the motor neurons involved in the pathogenesis of ALS (88). At the same time, the reduced ability to promote intestinal dysfunction affects the functioning of the brain–gut axis and plays an important role in the pathophysiological function of the intestine, affecting the development of ALS (86). It is necessary to analyze intestinal autophagy in ALS patients to further explore its relevance to the pathogenesis of ALS, which will help us to develop new targets for ALS treatment strategies.

### 3.4. Gut microbiota dysbiosis and immunity in ALS patients

The gut microbiota and the gut immune system interact through the GBA to help maintain immune tolerance and shape the immune response during inflammation (89). The maturation and function of CNS-resident macrophages and microglia are controlled by the gut microbiota (54). Alterations to the gut bacteria indirectly affect the ratio of macrophages to microglia, with M1 (pro-inflammatory) microglia producing ROS and pro-inflammatory cytokines such as NO, IL-1β, IL-6, TNF-α, and IFN-γ, and M2 (anti-inflammatory)-like microglial cytoplasm being associated with production of anti-inflammatory cytokines such as IL-4, IL-10, and IL-13 (28, 90). Rodrigues et al. (90) found that mSOD1 microglia may contribute to the progression of ALS and that SOD1^G93A^ increased the expression of the pro-inflammatory pattern recognition receptor RAGE, as well as releasing HMGB1- and SOD1-rich exosomes that promote pro-inflammatory responses. In addition, activation of TLR4 due to changes in gut microbiota composition and gut permeability further leads to the release of pro-inflammatory cytokines, endotoxins (LSP) (67), etc., from astrocytes and from the microglia themselves. LPS may alter gut homeostasis, inflammation, and permeability (91, 92) while activating various immune cells, including macrophages, neutrophils, and dendritic cells. Once activated (93), these cells produce a variety of pro-inflammatory cytokines, such as IL-2, IL-1β, IL-6, and TNF-α, which then cross the BBB into the brain via diffusion and cytokine transporters. These pro-inflammatory cytokines act on neuronal and glial cell-expressed receptors such as IL-1β, IL-6, and TNF-α, leading to neuroinflammation and neuronal death (94). Astrocytes (95), the most abundant glial cells in the nervous system, are important immune cells that play a key role in maintaining the BBB and regulating immune cell traffic and pro-inflammatory responses; they are widely found to be present in the postmortem brain tissue, cerebral white matter, and spinal cord of sALS and fALS patients. Gomes et al. (96) found that cortical astrocytes isolated from SOD1^G93A^ mouse model in the early stages of the disease exhibited neuroprotective features, with reduced cell proliferation, low expression of NF-κB and micro(miR)-146a, and weakened pro-inflammatory pathways, indicating impaired immune function in ALS patients. Changes in the gut microbiota also occur in ALS: in a controlled observational study of 37 ALS patients and 29 healthy BMI- and age-matched family members, Blacher et al. (30) found significant differences in gut flora and overall bacterial gene content between the ALS patients and the healthy controls. In most patients, stool analysis revealed the presence of inflammatory biomarkers, including fecal secretion of IgA, calcitonin, and eosinophil X. The presence of circulating endotoxins and activation of the immune system suggest disruption of the intestinal barrier and local inflammation in ALS. In a study of six ALS patients by Fang et al. (25), the fecal microbiota of ALS patients was noted to exhibit reduced *Firmicutes/Bacteroidetes* in proportion to an increase in the abundance of *Dorea* and a concomitant decrease in the abundance of *Oscillibacter, Anaerostipes*, and *Lachnospiraceae*. It has been found that most patients exhibit a decrease in microbiota diversity, including a decrease in the Firmicutes/Bacteroidetes (F/B) ratio, which is an indicator of dysbiosis. Changes in the F/B ratio have been found to be associated with various disorders, such as irritable bowel syndrome and inflammatory bowel disease (97), as well as with neurodegenerative diseases, such as PD, AD, and ALS (98). Several studies have shown that neuroinflammation is associated with the progression of ALS and that the microbiota, as a modifier of neurodegenerative disease risk (25), is closely associated with the inflammatory immune response. Given its role as the beginning of the GBA, further investigation of the corresponding immune mechanisms is warranted.

## 4. Potential strategies for treatment of ALS—How to exploit the GBA?

### 4.1. Improvements to intestinal microecology and repair of the intestinal barrier

It has been shown that altering the gut microbiota has clinical efficacy in the treatment of neurodegenerative diseases (99); gut-centered therapies include dietary interventions, next-generation probiotics, and fecal microbial transplants.

#### 4.1.1. Probiotic therapy

In the early 20th century, Nobel laureate Elie Metchnikoff proposed the concept of probiotics as “life” (3). The modern definition of probiotics is now widely recognized as “live microorganisms that when administered in adequate amounts confer a beneficial health effect on the host.” Probiotics are considered to be dietary factors that can influence the human intestinal microbiota, which can affect the composition and structure of the intestinal flora; which, in turn, plays a role in protecting the intestinal epithelial barrier, inhibiting pathogen production, and regulating the immune system (3). Studies have shown that the composition and products of the intestinal flora have a strong influence on immune response (16), and that the central nervous system is also influenced by the immune system (100). SCFAs, as important metabolites of the intestinal flora, can modulate the immune response (38) and act as important communication signals on the brain–gut axis (39). Through use of probiotics, the number of SCFA-producing microorganisms in the intestine can be increased (3). Abraham et al. (10) found that transgenic AD mice treated with probiotics exhibited better cognitive performance and a reduced number of Aβ plaques in the hippocampus compared to untreated AD mice. Akbari et al. (101) found that supplementation of *Lactobacillus acidophilus, Lactobacillus casei, Bifidobacterium bifidum*, and *Lactobacillus fermentum* improved cognitive function in patients with AD through facilitation of the normal functioning of the GBA pathway. Similarly, in a prospective longitudinal study of patients with ALS, Di Gioia et al. (102) found that intestinal flora was restored by probiotic supplementation compared to normal controls.

Zhang et al. (72) found that treatment of ALS mice with oxalate extended their life span by an average of 38 days. This demonstrates the effectiveness of oxalate, a natural bacterial product, in restoring microbial and intestinal homeostasis. Additionally, treatment of ALS mice with butyrate resulted in a significant decrease in the percentage of abnormal Paneth cells in the intestine. This treatment also restored the levels of lysozyme 1 and antimicrobial peptide defensin 5α in the intestine, as well as improving and slowing pathological progression of the intestine. This indicates the important role of butyrate in maintaining the physiological integrity of the intestine. Yang et al. (103) found that treatment with probiotic double immuno-galactooligosaccharide (Bimuno^®^ galactooligosaccharide; B-GOS) alleviated neuroinflammation and cognitive function in rats and greatly inhibited microglia activation, as well as expression of iNOS, CD68, CD32, SOCS3, and IL-6 expression. The use of symbiotic *A. muciniphila* supplementation alleviated ALS symptoms, while *Ruminococcus torque* and *P. distasonis* exacerbated these symptoms in Tg mice (74). Probiotic-4 (which contains *Lactobacillus, Bifidobacterium, Typhimurium*, and *Eosinophilus*) has been shown to attenuate aging-related disruptions in the integrity of the blood–brain barrier and the intestinal barrier; in plasma and brain LPS levels; and in IL-6, TNF-α, TLR4, and NF-κB translocation in the brains of aged mice (104). New research is now emerging on the use of synthetic bacterial therapies (105), in which engineered probiotics can be specifically designed to transform toxic metabolites in the gut into non-toxic forms. In addition to the potential to restore microbial homeostasis, probiotics can be considered as a vehicle for delivery of neuroactive compounds, which function as vital links in the brain–gut axis. Thus, probiotics should be considered a possible therapeutic/preventive strategy for neurological disorders.

#### 4.1.2. Dietary interventions

Włodarek et al. (106) found that a ketogenic diet, which is high in fat, moderate in protein, and low in carbohydrates, has the potential to reduce neuronal degeneration, and that this specific type of nutritional intervention may provide neuroprotection. Goncharova et al. (107), found that supplementation of pharmacological treatment with vitamins and changing the diet to a ketogenic diet could reduce the rate of motor neuron degeneration in ALS patients. However, it is important to note that the approach to dietary selection must be individualized. Modifying the gut microbiota through the diet and altering the signaling pathway of the GBA from the bottom up can slow down the rate of disease progression. Chico et al. (108) supplemented the diets of ALS patients with curcumin, a non-flavonoid polyphenol, and observed no clear progression of the disease after 6 months of follow-up. Fitzgerald et al. (109) concluded that total carotenoid intake was associated the risk of ALS. Polyphenols are a class of compounds composed of a variety of molecules; several studies in animal models of ALS have shown that these have neuroprotective effects (110). An effective anti-ALS food or compound typically appears to have at least one of either anti-inflammatory or antioxidant properties, as oxidative stress and inflammation play an important role in neuronal degeneration (111). Changing the gut microbiome through the diet and changing the signaling pathways of the GBA from the bottom up can slow the rate of disease progression.

#### 4.1.3. Fecal microbiota transplantation

Fecal microbiota transplantation (FMT) (112) refers to the transplantation of functional flora from the feces of healthy humans into the gastrointestinal tract of patients in order to establish new intestinal flora, with the goal of treating either intestinal or extraintestinal diseases. Zhang et al. (113) transplanted intestinal flora from patients with major depression into the intestine of GF mice and found that the mice developed depression-like behavior. Lu et al. (114) reported the case of a woman with ALS who was found at 12-month follow-up to have benefited from a washed microbiome transplant (WMT), an improved form of FMT, via a trans endoscopic bowel tube. The patient later suffered an unexpected scalp trauma, which was treated with prescribed antibiotics, leading to worsening of her ALS. The subsequent use of WMT rapidly improved this situation, successfully halting the progression of the disease. Based on current findings (115), FMT has the potential to play a role in treatment of neurological disorders such as ALS. The relatively low cost and low risk of FMT necessitates the study of mouse models of ALS for further exploration of this developing field.

The gut microbiota plays a key role in the neuropathogenesis of CNS-related diseases by directly or indirectly affecting the function of the GBA. Improving intestinal ecology through methods such as probiotic supplementation, dietary intervention, and FMT can help restore the intestinal ecology in ASL patients and improve the normal physiological functioning of the GBA. This may represent a new treatment strategy for central nervous system-related diseases such as ALS.

### 4.2. Antibiotic treatment

Antibiotics have recently found new uses in the treatment of NDs (116). In particular, beta-lactams (cephalosporin antibiotics) have been shown to be effective in the treatment and alleviation of ND symptoms and in the treatment of experimentally induced neurological disorders such as PD, AD, ALS, traumatic brain injury (TBI), epilepsy, cerebral ischemia, and neuropathy (84, 117, 118). CFT has been reported to have neuroprotective and anti-inflammatory effects, to protect neurons from oxidative stress and ionizing radiation, and to act as a free radical scavenger (84). Correlations between ALS and environmental effects have been found to suggest that SOD-1-Tg mice tend to exhibit virus-dependent biolysis and abnormal metabolite patterns during the prodromal phase when treated with broad-spectrum antibiotics (119). The results of this study suggest a direct correlation between viruses exposed to antibiotic treatment in SOD-1-Tg mice and certain bacterial species, particularly mucinophilic granulocytes that alleviate the symptoms of ALS and (*Erythrocyte torque* and *parabacteria*), which exacerbate the symptoms of ALS. Mice treated with the mucophilic granulocyte Akkermansia have also been found to show accumulation of nicotinamide in the central nervous system. Nicotinamide has recently been investigated to determine its relevance in improving ALS symptoms and thus motor function (120).

Bacterial metabolites, also known as post-antibiotics (121) or postbiotics, were treated with the bacterial product butyrate, an SCFA, in a study with SOD1^G93A^ mice (50). Butyrate treatment improves intestinal integrity and microbial homeostasis, acting along the brain–gut axis, and was found to have beneficial effects in SOD1^G93A^ mice in terms of extending the lifespan and delaying the progression of ALS.

### 4.3. Targeted therapies

Targeted therapies have gained popularity in recent years, and novel treatment options have become available, including phage therapy, carbon nanoparticles, and intestinal mucosal resurfacing. Further research on these approaches may hold promise for application in the treatment of ALS.

#### 4.3.1. Phage therapy

Phages (121) are viruses that specifically infect and kill particular intestinal bacterial pathogens. Unlike antibiotics, phages do not induce drug resistance (122). Ghadge et al. (123) used phage-specific raised single-chain variable fragment antibodies (scFvs) against SOD1^G93A^ in mice and found that these antibodies reduced motor neuron loss, microgliosis, astrocytosis, and SOD1 burden and aggregation. Improvements were observed in the aggregation and *in vitro* toxicity of mutant SOD1. Federici et al. (124) demonstrated that oral compound phage-targeted therapy can inhibit human intestinal microbiota symbiosis, improve intestinal inflammation, and effectively avoid antibiotic resistance. It can be speculated that the use of “phage cocktail” therapy or oral phage-targeted drugs to enhance SOD1 antibodies or antibody mimetics may be an effective treatment option for fALS (75, 76).

#### 4.3.2. Carbon nanoparticles

Carbon nanoparticles (125) have been shown to have a high adsorption capacity for bacterial toxins such as LPS. They are currently used in patients with advanced cirrhosis to improve their intestinal microbiota and are a new strategy for counteracting the biodivision and translocation of bacterial-derived products.

#### 4.3.3. Intestinal mucosal resurfacing

Intestinal mucosal resurfacing (124) is a technique that targets the duodenal surface in order to improve the intestinal barrier. This technique is currently used in patients with type 2 diabetes to improve the proliferation of enteroendocrine cells and endocrine cells, ultimately improving blood glucose levels.

ALS is a multifactorial syndrome as opposed to a single disease (115). Clinical treatment of various targets via the intestinal microbiota and its metabolites, including treatments aiming to improve the intestinal mucosal barrier and alter intestinal permeability, has the potential to treat ALS. Intestinal microbiota-mediated phage therapy can reduce both intestinal inflammation and neuroinflammation, decrease the accumulation of abnormal protein in motor neurons, reduce apoptosis, promote autophagy, and inhibit neuroinflammation. Therefore, intestinal mucosal resurfacing may be a promising therapy for ALS via the mechanisms of improving the gut microbiota and affecting GBA communication. Specific schemes are summarized in Table 1.

**Table 1:** Treating ALS via the GBA.

**Treatment plan**	**Detailed method**	**References**
Probiotic therapy	1. Restoration of microbial and intestinal homeostasis in ALS mice treated with oxalate	(72)
	2. In ALS mice treated with butyrate, the percentage of abnormal Paneth cells decreased significantly, and lysozyme 1 and antimicrobial peptide defensin 5α were restored in the intestinal tract	(72)
	3. Neuroinflammation was relieved and cognitive function improved with use of B-GOS in rats	(103)
	4. Probiotic-4 alleviates age-related damage to the integrity of the blood–brain barrier and intestinal barrier	(104)
	5. Curcumin supplementation for ALS patients resulted in absence of clear progression of the disease	(108)
Dietary intervention	1. A ketogenic diet reduces neuronal degeneration	(106)
	2. A ketogenic diet adds vitamins and reduces the rate of motor neuron degeneration	(107)
	3. No significant disease progression was observed in ALS patients whose diets were supplemented with curcumin	(108)
	4. Total carotenoid intake was associated with a reduced risk of ALS	(109)
	5. Polyphenols have neuroprotective effects	(110)
FMT	Use of WMT to delay disease progression	(114)
Antibiotics	1. Ceftriaxone has neuroprotective and anti-inflammatory effects	(84)
	2. Post-antibiotic treatment prolongs the lifespan of SOD1^G93A^ mice	(119)
Phage therapy	1. Phage-specific enhancement of single chain variable fragment antibodies (scFvs) against SOD1^G93A^ motor neuron loss, microgliosis, astrocytosis, and SOD1 burden and accumulation in mice	(123)
	2. Oral phage combination improves intestinal inflammation and effectively avoids antibiotic resistance	(124)
Carbon nanoparticles	High adsorption capacity for LPS etc	(125)
Intestinal mucosa resurfacing	Improvement of the intestinal barrier	(124)

## 5. Conclusion

ALS is a progressive and ultimately fatal disease that affects the motor neurons. The gut microbiota plays a key role in the pathogenesis of ALS by directly or indirectly altering GBA function. The review presented in this article suggests the conclusion that neuroinflammation is induced by an increased burden of pathogenic microbes and metabolites, compromising the intestinal barrier and activating immune regulation. These resulting induction of immune dysfunction, deposition and propagation of misfolded proteins, and autophagic responses marked by chronic inflammation impair normal neurons and contribute to the development of ALS. We can treat ALS via the gut microbiota and related derivatives; such treatment approaches include improving the intestinal microecology and repairing the intestinal barrier through probiotic supplementation, dietary intervention, or fecal transplantation. The use of antibiotics, post-antibiotic therapy, and targeted therapeutic approaches such as phage therapy can improve the gut microbiota and impact the nervous system via the brain–gut axis, opening up a new therapeutic avenue. Given the wide variety of derivatives of the gut microbiota machinery and the complex interactions of these derivatives with the GBA, a promising strategy is to combine multiple approaches from the domains of metabolomics, metagenomics, transcriptomics, and proteomics in order to identify the bacterial genes involved in regulation and to validate their potential efficacy as treatments for GBA-related diseases. Thus, with mounting evidence demonstrating the feasibility of treating ALS through mechanisms such as modulation of the gut microbiota, it is becoming increasingly plausible that modulation of the GBA via the gut microbiota represents a new era in the treatment of ALS.

## Author contributions

CZ devised the study. WW and XL were involved in the conception of the study and critically revised the manuscript. DH and LZ were involved in writing the article. All authors agreed to be accountable for the content of this article.
